# The BAM‐GelMA‐ADSCs bilayer patch promotes tissue regeneration and functional recovery after large‐area bladder defects in beagles

**DOI:** 10.1002/btm2.10745

**Published:** 2025-01-15

**Authors:** Ziyan An, Pengchao Wang, Zhengyun Ling, Kaipeng Bi, Zheng Wang, Jinpeng Shao, Jian Zhao, Zhouyang Fu, Meng Huang, Wenjie Wei, Shuwei Xiao, Jin Zhou, Weijun Fu

**Affiliations:** ^1^ Department of Urology Third Medical Center, PLA General Hospital Beijing China; ^2^ Medical School of PLA Beijing China; ^3^ Department of Urology Fifth Medical Center, PLA General Hospital Beijing China; ^4^ Department of Urology Hainan Hospital of Chinese PLA General Hospital Sanya China; ^5^ School of Medicine, Nankai University Tianjin China; ^6^ Department of Urology 960th Hospital of PLA Jinan China; ^7^ Department of Pain Beijing Anzhen Hospital of Capital Medical University Beijing China; ^8^ Department of Urology Air Force Medical Center Beijing China; ^9^ Beijing Institute of Basic Medical Sciences Beijing China

**Keywords:** adipose‐derived stem cells, bladder acellular matrix, bladder reconstruction, bladder tissue engineering, GelMA, hydrogel

## Abstract

Previous studies of bladder tissue engineering simply seeded cells onto the surface of the material, which makes the cells lack protection and makes it difficult to face the complex in vivo environment. The gelatin methacryloyl (GelMA) hydrogel possesses outstanding biocompatibility and distinctive photo‐crosslinking characteristics and is capable of offering a suitable three‐dimensional growth environment for cells. This study explored the optimal concentration of GelMA for encapsulating adipose‐derived stem cells (ADSCs) and combined it with bladder acellular matrix (BAM) to create a tissue‐engineered bladder patch. Results indicated that 10% GelMA more effectively promoted ADSCs proliferation and spreading compared to 7.5% and 12.5% concentrations, which can offer a better extracellular matrix environment for cells. BAM performed as an excellent substrate with mechanical properties and stitchability similar to natural tissues. Animal experiments demonstrated that the encapsulated ADSCs in GelMA enhanced patch vascularization in vivo and BAM‐GelMA‐ADSCs tissue‐engineered bladder patch can repair large‐scale bladder defects in beagles and promote bladder tissue regeneration and functional recovery. This photocrosslinking hydrogel‐acellular matrix patch provides a protective semi‐controlled environment for ADSCs, supporting the growth and viability of encapsulated cells in vivo, while being easy to suture and preventing leakage, and has significant clinical potential.

**Translational Impact Statement**

Our research has developed a bi‐layered tissue‐engineered bladder patch, where the upper layer of photocrosslinked hydrogel serves as a nurturing bed for stem cell growth and differentiation, enabling them to exert enhanced repair effects. The lower layer of the acellular matrix acts as a suture substrate and anti‐leakage barrier, providing stable support during the repair process. This bi‐layered patch can enrich surgical options for clinicians and is expected to bring favorable repair outcomes for patients, demonstrating excellent translational application prospects.

## 1 INTRODUCTION

Tissue engineering technology presents a novel method for repairing organs and tissues, making it possible to address bladder defects caused by tumors, trauma, ectropion, and other diseases more effectively than simple in‐situ suturing or intestinal substitution. Unlike skin, oral tissues, or bone, the bladder's function as a hollow organ for urine storage necessitates the construction of a scaffold or patch with excellent biocompatibility and mechanical properties.^1^ This patch must prevent leaks, be stitchable, and possess stretchability and shrinkability to meet the bladder's physiological requirements.^2^ Furthermore, the tissue‐engineered bladder patch should exhibit good biodegradability to prevent the formation of bladder or urethral stones that can obstruct urine flow.^3^

Among different biomaterials, decellularized products derived from natural tissues offer inherent biocompatibility and the extracellular matrix (ECM) structure, along with endogenous growth factors, facilitating cell adhesion, proliferation, and differentiation, thereby accelerating regenerative healing. The bladder acellular matrix (BAM) is a prime example of such a material and stands out as an exceptional material for bladder repair.^4^ Previously, we had adhered adipose‐derived stem cells (ADSCs) to the BAM surface, demonstrating the effectiveness of BAM as a carrier for delivering ADSCs in repairing rat bladder defects.^5^ However, it was found that ADSCs on the surface of BAM often needed to face complex surgical and in vivo environments, lacking effective protection for the cells and resulting in a higher risk of detachment and death. Recent studies also have similar problems, highlighting the need for a better growth environment for the cells.^6^, ^7^

Hydrogels, a key element in tissue engineering, are widely used in various types of tissue repair, including skin, bone, and cartilage, due to their broad application prospects.^8^, ^9^, ^10^ The BAM hydrogel developed by Manzano could induce the differentiation of ADSCs into smooth muscle cells in vitro, but lacked in vivo validation.^11^ However, the bladder presents a more complex application scenario. Repair materials must withstand urine exposure, peritoneal friction, and the stretching and deformation caused by bladder contraction and relaxation.^12^ Currently, few hydrogels can handle these challenges, necessitating the combination of hydrogels with special substrate materials. Xiao previously prepared BAM hydrogel and combined it with a bilayer silk fibroin scaffold to construct a composite patch for rat bladder repair.^13^ The BAM hydrogel protected seed cells in vivo, while the silk fibroin (SF) membrane served as a suture substrate and urine barrier. However, this hydrogel could not form rapid and stable crosslinking, and the degradation of SF was prone to producing calculi. Therefore, it is necessary to explore novel hydrogels and substrate materials to construct better composite patches.

Gelatin methacryloyl (GelMA), created by polymerizing gelatin with methacrylic anhydride, possesses rapid photocrosslinking capabilities, significantly expanding its applications.^14^ Although GelMA is extensively used in tissue engineering, its application in bladder tissue engineering is limited.^15^ ADSCs are known for their excellent differentiation potential, wide availability, ease of extraction, and multilineage differentiation capabilities.^16^ The GelMA's loose network structure facilitates oxygen and nutrient exchange, creating an ECM‐like environment for ADSCs growth and differentiation.^17^ Moreover, GelMA contains RGD motifs that enhance cell adhesion and growth, and MMP motifs that promote cell remodeling, aiding in cell survival and proliferation.^18^ Researchers can adjust the hydrogel's degradation rate, mechanical properties, swelling behavior, and chemical characteristics to mimic natural ECM features, thus influencing stem cell fate.^19^, ^20^ Studies indicate that variations in GelMA concentration can alter the hydrogel's mechanical properties, affecting the differentiation of ADSCs into smooth muscle or osteogenic lineages.^21^, ^22^ Therefore, exploring the optimal concentration of GelMA and combining it with BAM represents an ideal approach for constructing tissue‐engineered bladder patches.

Additionally, the majority of current research on bladder tissue engineering is conducted using rat models. Although the data are promising, translating these findings into clinical applications is found to be challenging because rats have a bladder capacity of merely 1–2 mL, which differs significantly from that of humans.^23^, ^24^, ^25^ These size and volume variations will result in substantial discrepancies. However, the bladder capacity of adult beagles is 100–200 mL, being closer to that of teenagers which is 300 mL.^26^, ^27^ Further large animal studies are beneficial for determining the therapeutic effects of tissue‐engineered bladder patches for future clinical utilization.

**SCHEME 1:**
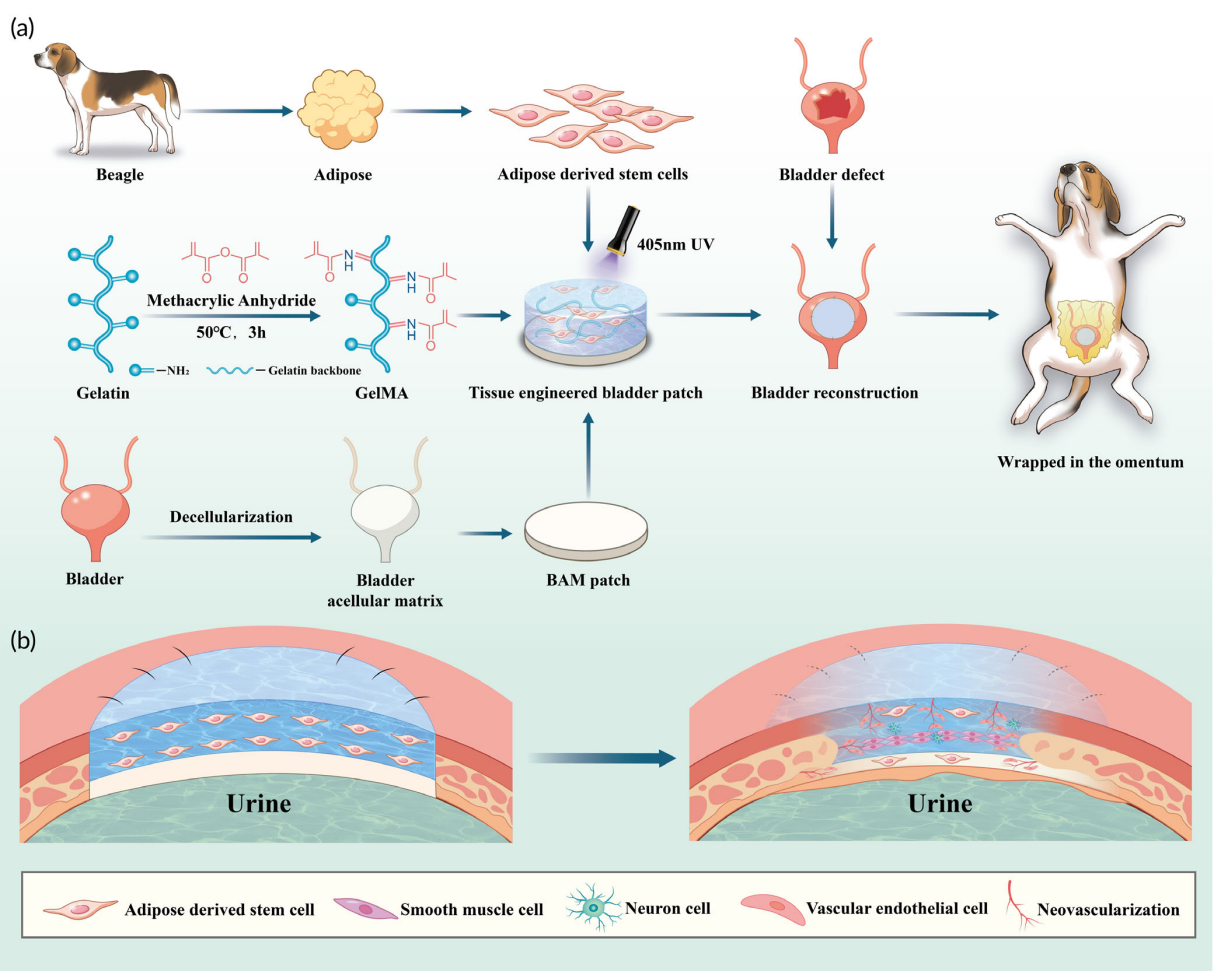
(a) Schematic illustration of preparation and application of BAM‐GelMA‐ADSCs bilayer patch. (b) BAM serves as the suture substrate and urine barrier, and GelMA provides a hotbed for the growth and differentiation of ADSCs, promoting bladder tissue regeneration and functional recovery.

Therefore, this study conducted mechanical property, swelling, degradation performance, and biocompatibility tests on three concentrations of GelMA to determine the optimal concentration for ADSCs encapsulating. Biological and mechanical characterization of BAM was also performed and evaluated from the perspective of clinical use and suturing. Subsequently, GelMA was mixed with ADSCs and seeded onto BAM to construct an ideal repair patch after photocrosslinking. Bladder reconstruction surgery was then performed in beagles, and the repair effect of tissue‐engineered bladder patches in vivo was evaluated through imaging, functional, and histological examinations (Scheme 1).

## 2 RESULTS

### 2.1 Synthesis of GelMA

**FIGURE 1:**
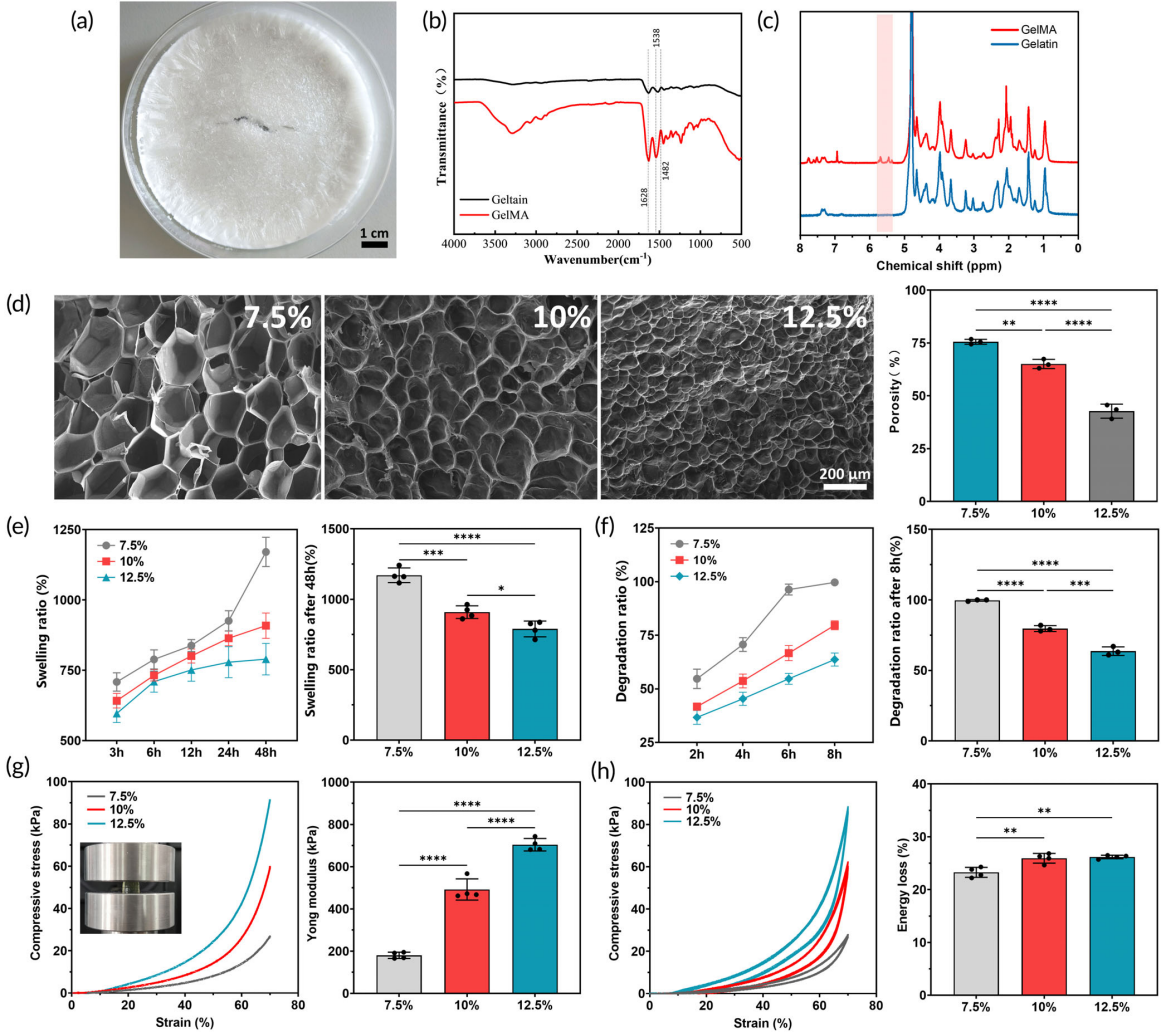
Characterization of GelMA. (a) Foam‐like GelMA. (b) FT‐IR (*n* = 3). (c) ^1^H NMR spectroscopy (*n* = 3). (d) Porosity observation by SEM (*n* = 3). (e) Swelling ratio of GelMA (*n* = 4). (f) Degradation ratio of GelMA (*n* = 12, 3 per time point). (g) Compression test (*n* = 4). (h) Compression‐cycle test (*n* = 4). (*p‐*values are calculated using the one‐way analysis of variance (ANOVA), *p < 0.05, **p < 0.01, ***p < 0.001, ****p < 0.0001.)

The synthesized GelMA exhibits a white, loose, foam‐like structure (Figure 1a). FT‐IR spectroscopy revealed characteristic peaks at 1628 cm^−1^ (amide I), 1538 cm^−1^ (amide II), and 1482 cm^−1^ (amide III) in the spectrum (Figure 1b). These peaks correspond to C=O stretching, C‐N‐C=O symmetry stretching, N‐H in‐plane bending, and C‐N stretching within the GelMA chemical structure, confirming the coupling of methacryloyl groups. Additionally, ^1^H NMR spectroscopy (Figure 1c) showed two new peaks at 5.45 ppm and 5.69 ppm in GelMA, corresponding to the alkenyl double bond (C=C) of the methacrylate group conjugated with gelatin, further verifying the synthesis of GelMA.^28^

### 2.2 Characterization of GelMA hydrogel

Scanning electron microscopy revealed that GelMA hydrogels possess uniformly distributed pores, with porosity rates of 75.58 ± 1.17% for 7.5% GelMA, 65.07 ± 2.19% for 10% GelMA, and 42.73 ± 3.34% for 12.5% GelMA (Figure 1d). Swelling experiments indicated that 12.5% GelMA reached swelling equilibrium at 48 h with a swelling ratio of 789.79 ± 56.00%, while 7.5% and 10% GelMA had swelling ratios of 1170.84 ± 51.97% and 908.71 ± 45.00%, respectively, showing an upward trend (Figure 1e). The higher water absorption and expansion of low‐concentration hydrogels may be due to their loose network structure and high porosity.

An in vitro enzymatic degradation test using type II collagenase demonstrated that the degradation rate decreases with increasing GelMA concentration. Specifically, 7.5% GelMA degraded over 95% (95.91 ± 2.52%) within 6 h and nearly completely (99.62 ± 0.43%) within 8 h. In contrast, the degradation rates of 10% and 12.5% GelMA at 8 h were 79.51 ± 2.04% and 63.74 ± 2.70%, respectively (Figure 1f).

The compressive stress–strain curves (Figure 1g) indicate a positive correlation between GelMA concentration and the mechanical properties of the scaffolds. The Young's modulus values for 7.5%, 10%, and 12.5% GelMA were 180.78 ± 15.0 kPa, 492.30 ± 50.14 kPa, and 704.04 ± 29.35 kPa, respectively, demonstrating enhanced mechanical properties with higher GelMA concentrations. All three hydrogel groups maintained structural integrity at 70% strain, exhibiting good deformability. Cyclic compression tests showed similar stress–strain curves for compression and recovery among the three hydrogel groups, indicating excellent fatigue resistance and self‐recovery capabilities. Notably, 7.5% GelMA exhibited the lowest energy loss, indicating superior fatigue resistance and self‐recovery ability (Figure 1h).

### 2.3 Characterization of BAM

**FIGURE 2:**
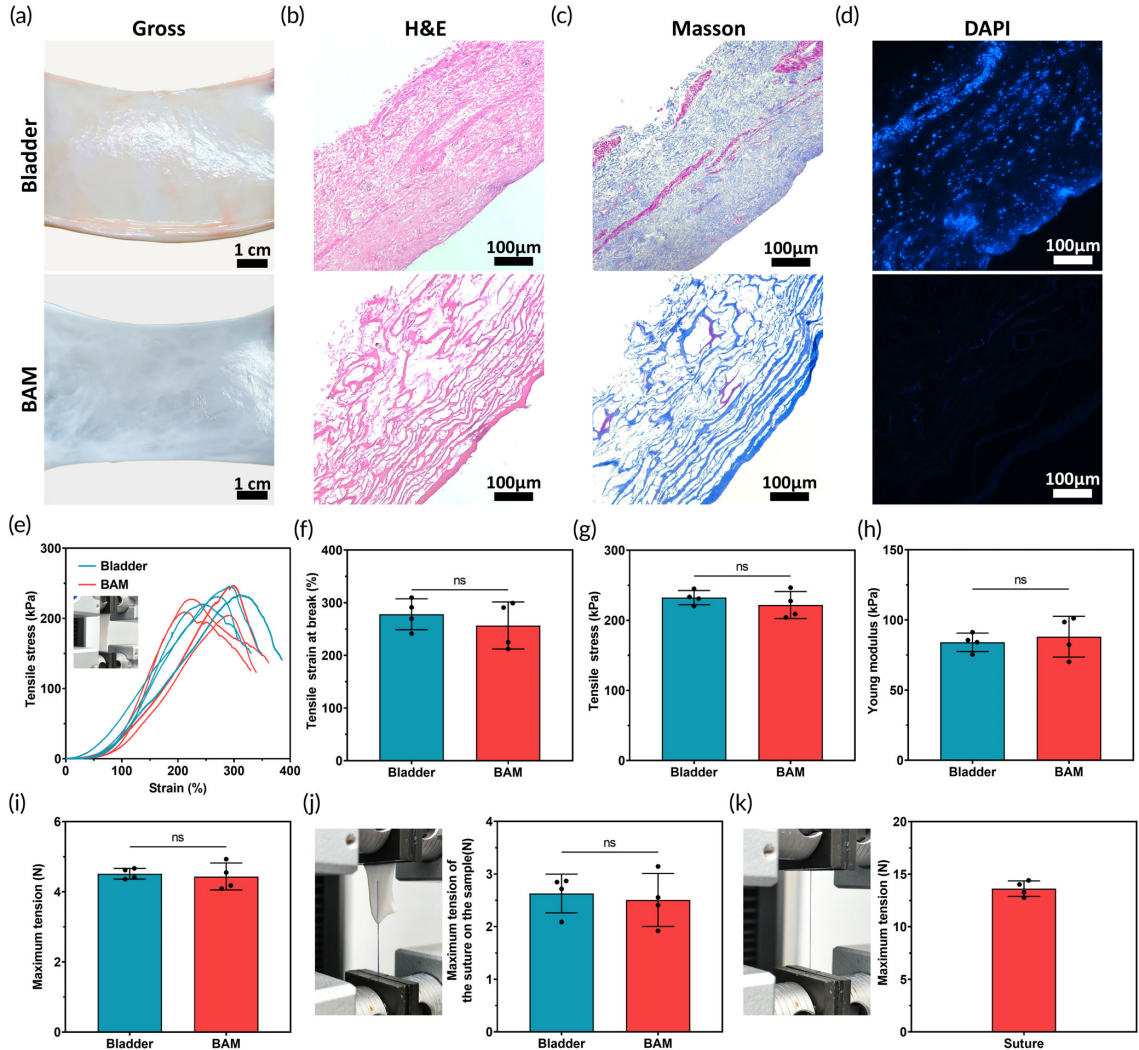
Histological staining and mechanical examination of BAM. (a) Natural bladder and BAM. (b) H&E staining. (c) Masson trichrome staining. (d) DAPI (*n* = 3). (e) Tensile‐stress curve of the bladder and BAM (*n* = 4). (f) Tensile strain at break. (g) Maximum tensile stress. (h) Young's modulus. (i) Maximum tension; (j) Maximum tension of the suture on the bladder and BAM (*n* = 4). (k) Maximum tension of the suture (*n* = 4). (*p‐*values are calculated using the paired *t*‐test, ns: No significant difference.)

Following decellularization, the color of BAM changed from pink to white (Figure 2a). H&E and Masson's trichrome staining revealed that BAM had lost a significant amount of cellular components, retaining only a loose collagen network structure beneficial for cell adhesion and growth. DAPI staining confirmed the absence of nuclear structures in the prepared BAM (Figure 2b–d). The DNA content of BAM (43.49 ± 3.68) was significantly lower than that of natural bladder mucosa (198.2 ± 7.63), indicating effective decellularization and suggesting a reduced risk of immune response after transplantation (Figure S1). Tensile tests showed that BAM's tensile strain at break (256.76 ± 44.71%), tensile stress (221.86 ± 19.31 kPa), Young's modulus (88.10 ± 14.54 kPa), and maximum tension (4.44 ± 0.38 N) were comparable to natural bladder tissue (278.06 ± 29.33%; 232.42 ± 10.08 kPa; 84.08 ± 6.58 kPa; 4.52 ± 0.15 N), with no significant differences (Figure 2e–i). Additionally, BAM's suturing performance as a patch using 5–0 absorbable sutures was tested. Results showed excellent cut resistance, with no significant difference in the maximum tension of the suture on the sample between the bladder (2.63 ± 0.37 N) and BAM (2.51 ± 0.50 N) (Figure 2j). The maximum tension the suture itself could withstand was 13.63 ± 0.73 N (Figure 2k).

### 2.4 Biocompatibility of GelMA and BAM

**FIGURE 3:**
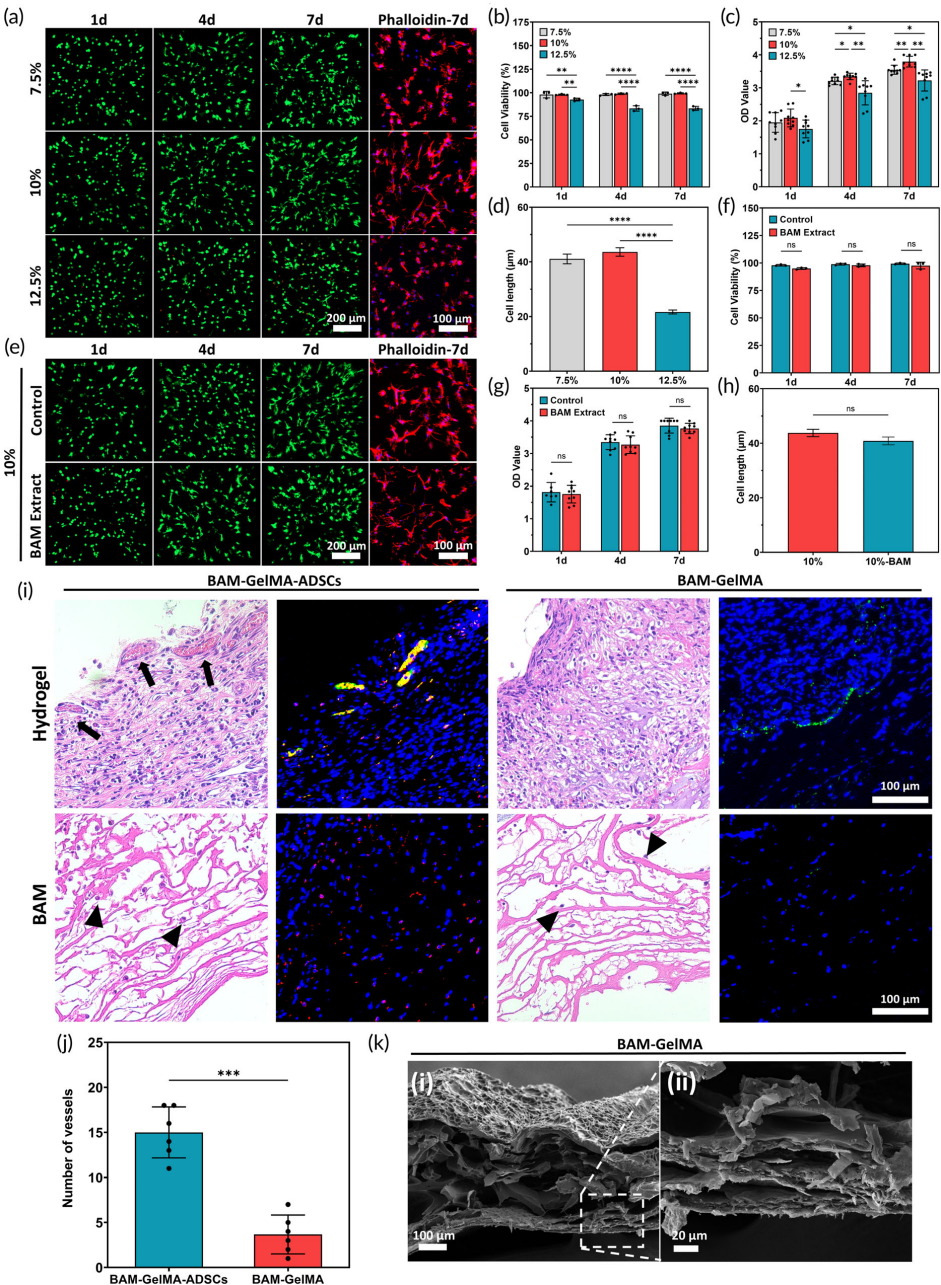
Biocompatibility of GelMA and BAM and in vitro and in vivo experiments of BGA and BG patches. (a) Live/dead staining and cytoskeleton staining of ADSCs in different concentrations of GelMA (*n* = 3). (b) Cell viability of ADSCs in GelMA. (c) Proliferation capacity of ADSCs in GelMA (*n* = 3). (d) Cell extension length of ADSCs in GelMA. (e) Live/dead staining and cytoskeleton of ADSCs in BAM‐GelMA (*n* = 3). (f) Cell viability of ADSCs in BAM‐GelMA. (g) Proliferation capacity of ADSCs BAM‐GelMA (*n* = 3) (*p‐*values are calculated using the two‐way ANOVA, *p < 0.05, **p < 0.01, ***p < 0.001, ****p < 0.0001, ns: No significant difference). (h) Cell extension length of ADSCs in BAM‐GelMA. (i) H&E staining and immunofluorescence staining of two patch groups (Nucleus: DAPI, blue; Blood vessels: PECAM1, green; CM‐DiL‐labeled ADSCs: Red; Black arrow: Regenerated vessels; Black triangle: Cells that migrated into the BAM) (*n* = 3). (j) Number of blood vessels in both patches (*p‐*values are calculated using the paired *t*‐test, ***p < 0.001, ns: No significant difference). (k) SEM of BAM‐GelMA patch.

Live/dead staining results showed that cell viability in the 7.5% and 10% GelMA groups exceeded 97% on days 1, 4, and 7. In contrast, the 12.5% GelMA group exhibited lower cell viability, with 92.76 ± 1.47%, 82.28 ± 2.83%, and 83.40 ± 2.12% on days 1, 4, and 7, respectively, showing statistically significant differences compared to the other groups (Figure 3a,b). CCK‐8 assay results indicated that cell proliferation in the 12.5% GelMA group was significantly lower than in the 7.5% and 10% groups on all days, consistent with the live/dead staining results. Notably, the 10% GelMA group demonstrated significantly higher cell proliferation than the 7.5% group on days 4 and 7 (Figure 3c). Additionally, cytoskeletal staining on day 7 revealed that cells in the 7.5% and 10% GelMA groups had better extensibility and spreading compared to the 12.5% GelMA group (Figure 3d). Figure S3 presented the growth of ADSCs in 10% GelMA. RT‐PCR results showed that ADSCs in 10% GelMA expressed higher levels of Oct4 and Sox2, which are crucial for maintaining the multi‐directional differentiation potential of mesenchymal stem cells (Figure S4).

BAM is an essential component of tissue‐engineered bladder patches. To verify BAM's biocompatibility, ADSCs encapsulated with 10% GelMA were cultured in both blank medium and BAM extract. Live/dead staining, CCK‐8 assay, and cytoskeletal staining showed no significant differences between the two conditions, indicating excellent biocompatibility of BAM (Figure 3e–h).

### 2.5 Construction of the BAM‐GelMA‐ADSCs bilayer patch

Scanning electron microscope (SEM) revealed that the BG patch has a bilayer structure, consisting of a porous hydrogel layer on top and a multilayered loose structure in the underlying BAM (Figure 3k). H&E staining of the in vivo embedded patch showed significant cell aggregation in the hydrogel area of both BGA and BG patches post‐omentum encapsulation, with cells located within the hydrogel structure. Notably, vascular‐like structures formed on the surface of the BGA patch, whereas such structures were scarce in the BG patch. DAPI staining indicated a significant presence of CM‐DiL‐labeled ADSCs within both the hydrogel and BAM of the BGA group, confirming the survival and proliferation of ADSCs encapsulated by GelMA in vivo. Additionally, a small number of cell nuclei in the BAM of both patches suggest that the loose BAM structure attracts ADSCs from the hydrogel and cells from the animal's body to migrate into it. Immunofluorescence staining showed higher expression of the vascular endothelial marker PECAM1 in the BGA patch after omentum encapsulation compared to the BG patch (Figure 3i). The BGA patch had a significantly higher number of regenerated blood vessels (15.00 ± 2.83) compared to the BG group (3.67 ± 2.16) (***p < 0.001) (Figure 3j).

### 2.6 Animal surgery and postoperative imaging examination

**FIGURE 4:**
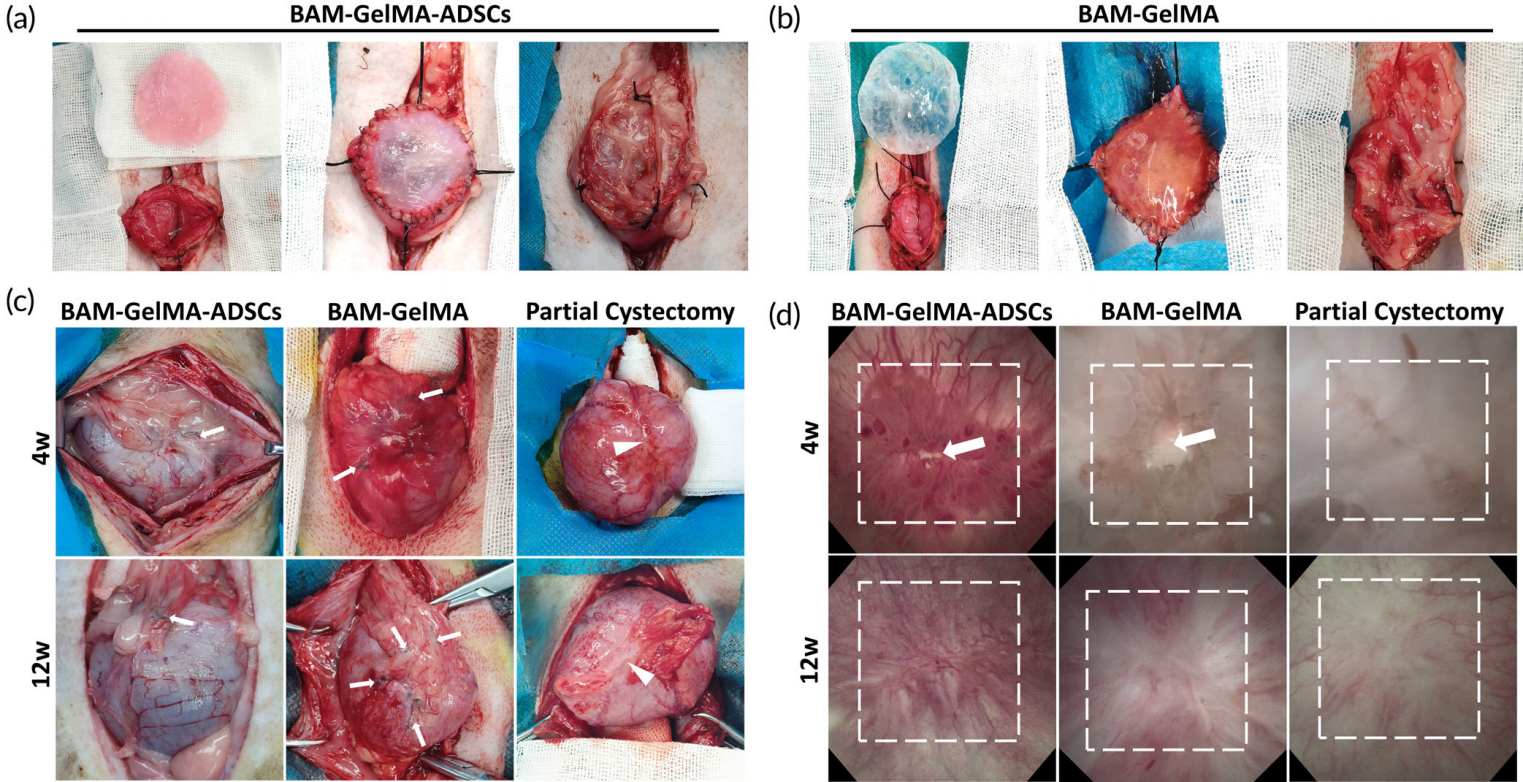
Tissue engineering bladder patch repair surgery and postoperative morphological examination. (a) BGA patch. (b) BG patch. (c) Postoperative morphological observation (white arrow: Suture; white triangle: Suture scar). (d) Postoperative cystoscopy (white square: Repaired area; white arrow: BAM). (*n* = 6, 3 per time point).

All animals survived normally after surgery, although three developed incision infections shortly after surgery, which improved with treatment. As shown in Figure 4c, the bladder repair area in the BGA group was covered with omental fat at 4 and 12 weeks, exhibiting color and morphology similar to the normal bladder without obvious scar contracture. In the BG group, the repair area was also covered with omentum at 4 weeks but appeared redder, indicating some degree of fibrosis, which lessened by 12 weeks. In the partial cystectomy (PC) group, distinct suture scars were visible on the bladder surface at both 4 and 12 weeks.

Cystoscopy revealed extensive follicular hyperplasia in the repair area of both BGA and BG groups at 4 weeks postoperatively. In the BGA group, most patches were covered by follicles and urothelium, while in the BG group, some BAM remained exposed. The PC group showed a distinct longitudinal incision at 4 weeks. By 12 weeks, the BGA group's repair area resembled normal bladder mucosa, with a smooth surface, abundant blood supply, and no significant follicles. In the BG group, distinct scars with relatively few blood vessels and some residual follicles were observed on the repair area surface. The PC group displayed a bloodless scar at the sutured site (Figure 4d). No abnormalities, such as bladder stones or diverticula, were found in any animals.

**FIGURE 5:**
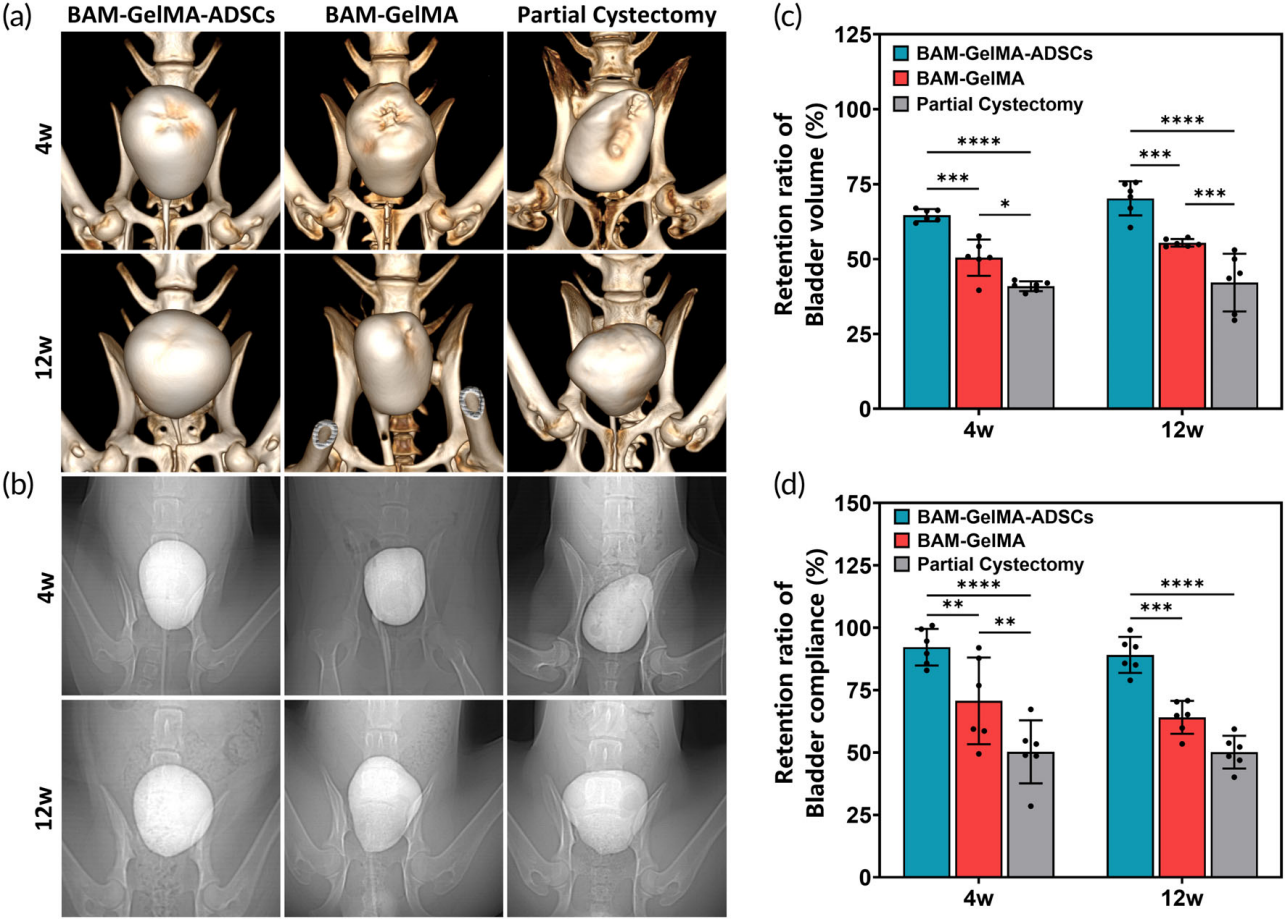
Postoperative imaging and urodynamic examination. (a) Three‐dimensional CT reconstruction of the bladder. (b) Cystography. (c) Urodynamic examination of bladder volume retention ratio (postoperative/preoperative). (d) Bladder compliance retention ratio (postoperative/preoperative). (*n* = 6, 3 per time point) (*p‐*values are calculated using the two‐way ANOVA, *p < 0.05, **p < 0.01, ***p < 0.001, ****p < 0.0001).

At 4 weeks postoperatively, bladder CT three‐dimensional reconstruction images revealed well‐maintained overall morphology in the BGA group, with minor indications of patch repair. The BG group exhibited significant contraction and wrinkling of the patch in the repair area. A distinct longitudinal suture scar was visible in the middle of the bladder in the PC group. By 12 weeks, the BGA group's bladder had nearly returned to normal, with a smooth surface in the repair area. In contrast, the BG group still showed clear signs of patch repair. The PC group displayed a healing scar at the top of the bladder (Figure 5a). Cystography showed that no urine leakage occurred in the three groups at 4 weeks and 12 weeks (Figure 5b).

### 2.7 Urodynamic examination

In terms of bladder capacity, the bladder volume retention ratio at 4 weeks postoperatively was highest in the BGA group (64.66 ± 2.03%), compared to the BG group (50.47 ± 6.08%) and the PC group (40.94 ± 1.65%) (***p < 0.001, ****p < 0.0001). The BG group also showed a higher bladder volume retention ratio than the PC group (*P < 0.05). At 12 weeks postoperatively, the BGA group maintained the highest bladder volume retention ratio (70.25 ± 5.69%), followed by the BG group (55.40 ± 1.27%) and the PC group (42.16 ± 9.60%) (***p < 0.001, ****p < 0.0001). The BG group continued to outperform the PC group in bladder volume retention (***p < 0.001) (Figure 5c).

Regarding bladder compliance, at 4 weeks postoperatively, the BGA group exhibited the highest bladder compliance retention ratio (92.22 ± 7.32%), followed by the BG group (70.69 ± 17.36%) and the PC group (50.26 ± 12.63%) (**p < 0.01, ****p < 0.0001). The BG group's compliance retention was also superior to the PC group's (**p < 0.01). At 12 weeks postoperatively, the BGA group again showed the highest bladder compliance retention ratio (89.11 ± 7.20%), with the BG group (64.08 ± 6.59%) and the PC group (50.16 ± 6.58%) trailing behind (***p < 0.001, ****p < 0.0001) (Figure 5d).

### 2.8 Histological examination

H&E and Masson's trichrome staining at 4 weeks postoperatively revealed abundant collagen fibrous tissue and scattered smooth muscle tissue in the repair area of the BGA group. The BG group exhibited severe fibrosis, significant inflammatory cell infiltration, and an absence of smooth muscle tissue. Both groups showed follicular urothelial hyperplasia in the urothelium and incompletely degraded sutures in the repair area.

**FIGURE 6:**
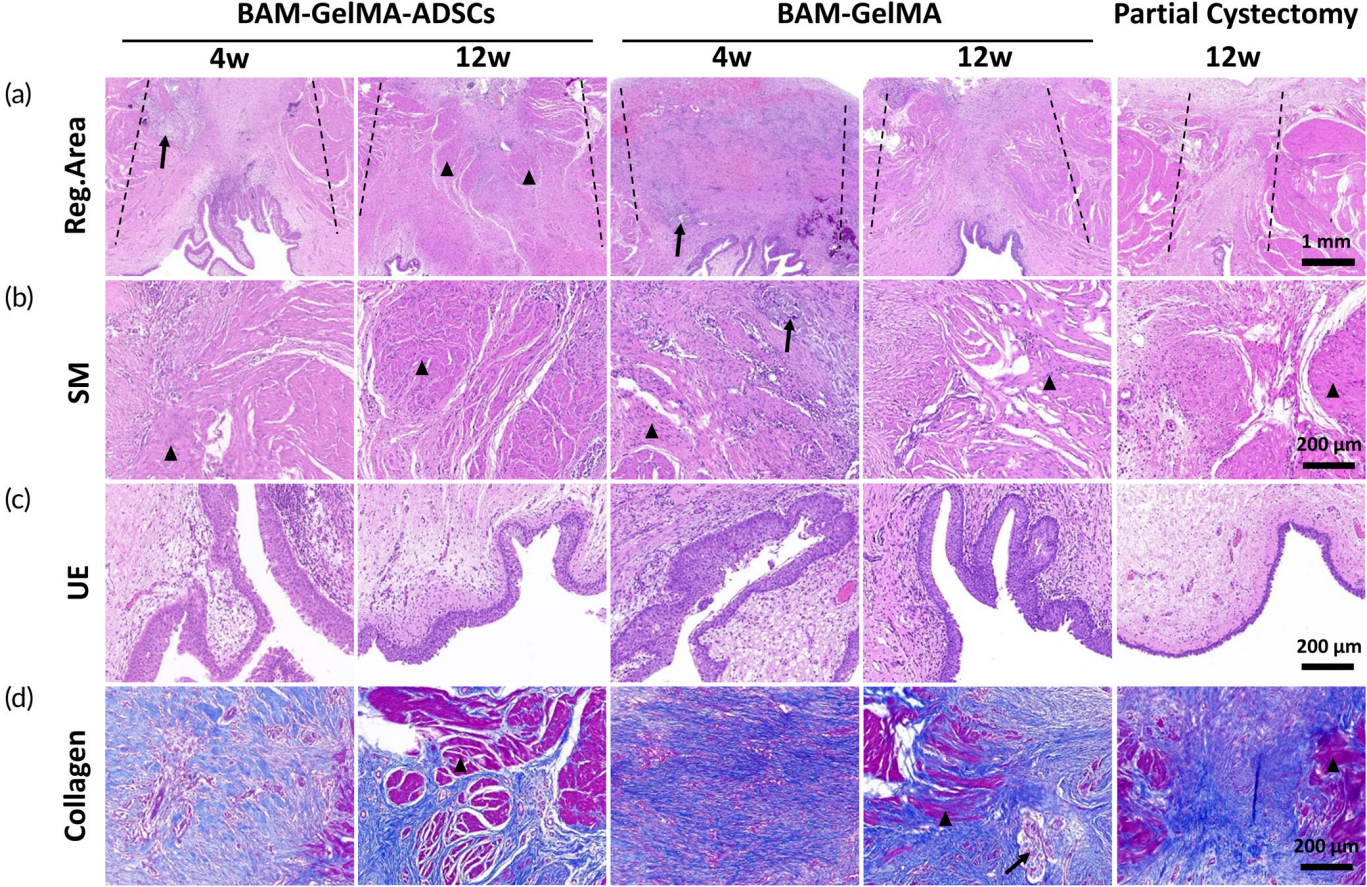
Histological examination of the regenerated bladder wall in the BAM‐GelMA‐ADSCs, BAM‐GelMA, and partial cystectomy group. (a) H&E staining of regenerated area in different groups. (b) H&E staining of the smooth muscle layer. (c) H&E staining of the urothelial layer. (d) Masson's trichrome staining of collagen tissue in bladder regeneration area (black dashed line: Edge of repair area; black arrow: Sutures; black triangle: Regenerate muscle) (*n* = 6, 3 per time point).

At 12 weeks postoperatively, the BGA group's repair area displayed significant regenerated smooth muscle surrounded by collagen fibrous tissue and some vascular regeneration. The BG group showed a reduced inflammatory response compared to 4 weeks, with extensive collagen fibrous hyperplasia and a small amount of regenerated muscle in the repair area. In the PC group, the healing area contained less regenerated muscle tissue, predominantly composed of collagen fibrous hyperplasia. Urothelial cell proliferation and the formation of a multilayered structure were observed in all three groups (Figure 6a–d).

**FIGURE 7:**
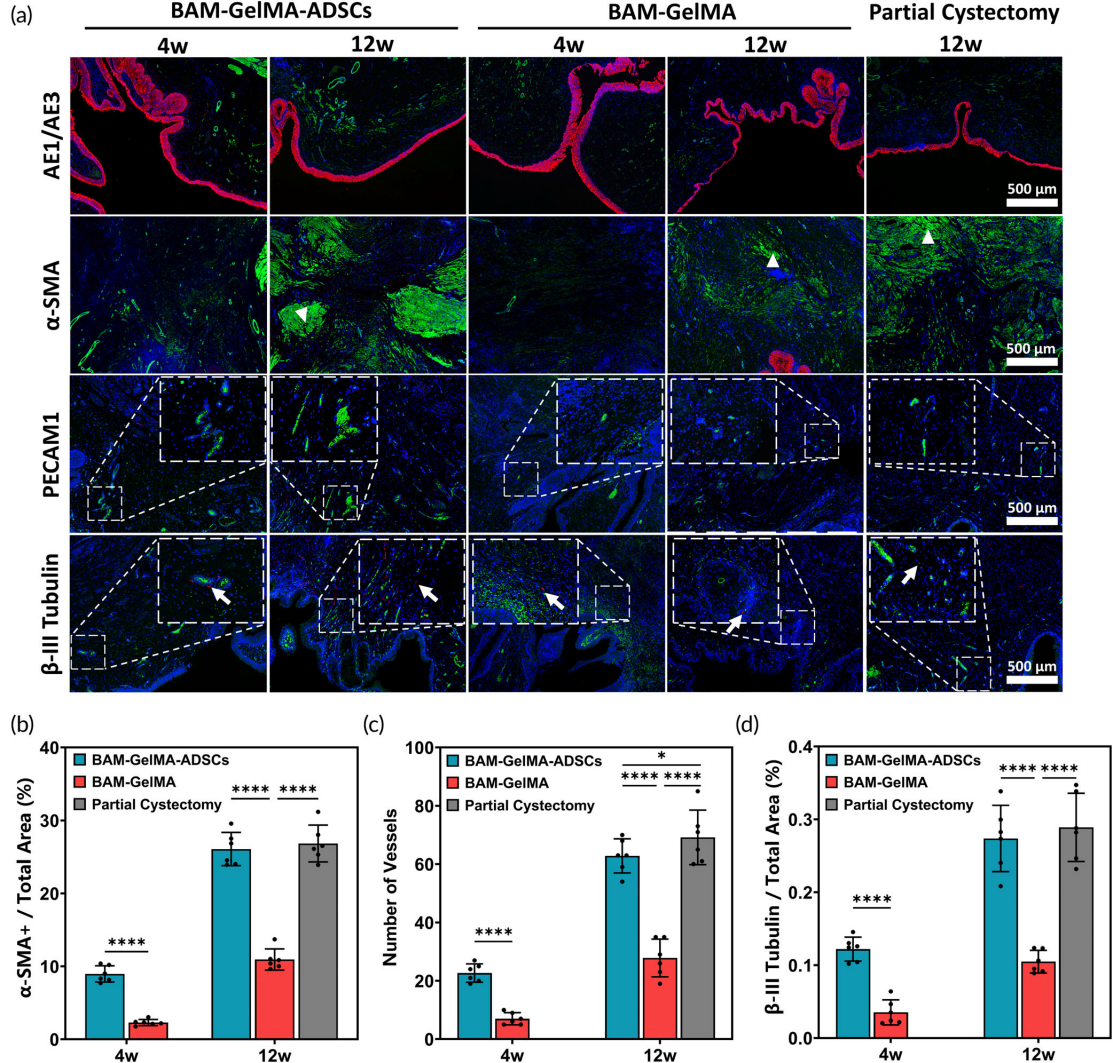
Immunofluorescence assessment of the regeneration of smooth muscle, innervation, and angiogenesis. (a) AE1/AE3: Urothelium, red; α‐SMA: Smooth muscle, green; PECAM1: Blood vessels, green; β‐III Tubulin: Neurons, red; DAPI: Nucleus, blue; white triangle: Regenerate muscle; white arrow: Neurons. (b) Area of α‐SMA positive. (c) Number of PECAM1‐positive vessels. (d) Area of β‐III Tubulin positive (*n* = 6, 3 per time point) (*p‐*values are calculated using the two‐way ANOVA, *p < 0.05, ****p < 0.0001).

Immunofluorescence staining at 4 weeks postoperatively showed that the BGA group had higher levels of α‐SMA positivity (8.97 ± 1.11%), PECAM1‐positive blood vessels (22.67 ± 3.14), and β‐III Tubulin positivity (0.12 ± 0.02%) compared to the BG group (2.31 ± 0.42%, 7.00 ± 2.10, and 0.04 ± 0.02%, respectively) (****p < 0.0001 for all comparisons) (Figure 7a). At 12 weeks, the area ratio of α‐SMA positivity was similar between the BGA group (26.07 ± 2.28%) and the PC group (26.84 ± 2.52%) (*p* > 0.05), both higher than the BG group (10.94 ± 1.45%) (****p < 0.0001) (Figure 7b). The BGA group had more PECAM1‐positive blood vessels (62.83 ± 5.85) than the BG group (25.67 ± 8.33) (****p < 0.0001), but fewer than the PC group (69.17 ± 9.35) (*p < 0.05) (Figure 7c). The area ratio of β‐III Tubulin positivity showed no significant difference between the BGA group (0.27 ± 0.05%) and the PC group (0.29 ± 0.05%) (*p* > 0.05), with both being higher than the BG group (0.11 ± 0.02%) (****p < 0.0001) (Figure 7d).

## 3 DISSCUSSION

Our previous studies have demonstrated the efficacy of BAM in rat bladder repair and have also fabricated a bilayer patch by employing BAM hydrogel and SF.^5^, ^13^ However, the BAM hydrogel lacks the capacity for rapid and stable crosslinking, and the degradation of SF is prone to generating stones. Due to the inherent properties of the materials, this patch might be incapable of repairing larger bladder defects. GelMA is widely used in tissue engineering due to its photocrosslinking properties and well processability but rarely applied in bladder tissue engineering.^29^, ^30^ Therefore, in this study, we utilized GelMA to offer a three‐dimensional growth space for cells and further investigated the optimal concentration of its encapsulation of ADSCs, enabling it to provide a protective environment for cells in vivo. Subsequently, GelMA was combined with BAM to construct the BAM‐GelMA‐ADSCs bilayer patch. In the loose structure of GelMA, ADSCs endowed the patch with a better vascularization capability. Furthermore, the bilayer patch can suture repair the bladder defect of beagles and facilitate the regeneration of bladder tissue and functional recovery.

Variations in hydrogel concentrations result in diverse porosity, swelling capacity, degradation rates, and mechanical properties, significantly impacting cellular viability, function, and even differentiation.^20^, ^29^, ^31^ In this study, GelMA at concentrations of 7.5%, 10%, and 12.5% (w/v) all possessed porous structures, and the higher the GelMA concentration, the denser the internal structure and the smaller the pores. The porous structure better provides channels for the exchange of oxygen, carbon dioxide, and nutrients.^32^ Therefore, hydrogels with higher porosity extend adequate nutritional support to cells and also offer a suitable microenvironment for cell adhesion, proliferation, differentiation, and tissue remodeling, ultimately promoting angiogenesis under physiological conditions.^33^, ^34^ Furthermore, hydrogel porosity directly correlates with mechanical properties and stiffness, with higher porosity leading to increased pliability.^35^ Compression experiments confirmed this trend, showing that higher concentration GelMA exhibited a greater Young's modulus and resilience to compressive stress under identical deformation conditions. This enhanced mechanical performance is advantageous for surgical manipulation and implantation. Compression cycling experiments further investigated GelMA's resilience, revealing exceptional fatigue resistance and self‐recovery capabilities. This self‐recovery ability is highly compatible with the physiological expansion and contraction activities of the bladder.^36^ However, the higher GelMA concentrations tend to inhibit cell spreading and proliferation. The 12.5% GelMA in our study showed significantly more dead cells, likely due to its low porosity and high network density, which restricted nutrient and oxygen exchange.

The expansion capability and degradation rate are pivotal factors for superior hydrogels, influencing cell proliferation and wound healing.^37^, ^38^ Swelling experiments revealed that low‐concentration GelMA exhibited pronounced swelling, facilitating higher water penetration into the hydrogel network and creating a more spacious milieu for cell spreading. This swelling capacity also allows hydrogels to absorb excess wound fluid or release moisture as needed, maintaining optimal wound hydration.^39^ However, excessive swelling can compromise mechanical integrity, making highly swollen hydrogels more prone to fragmentation, thus necessitating the identification of an optimal swelling ratio. For in vivo implantation, the degradation rate is crucial. Ensuring sufficient cell proliferation and viability before complete hydrogel degradation is essential for optimal reparative and regenerative outcomes. Collagenase significantly accelerated the degradation process, with 7.5% GelMA degrading the fastest, potentially limiting its ability to sustain a cell growth environment for cell proliferation in vivo. Conversely, in vivo degradation studies by Jing He have shown that 10% GelMA, which balances swelling capacity and degradability, may be more suitable for in vivo experiments, as 5% GelMA degraded rapidly in rats, with the fluorescent signal disappearing within 2–3 weeks, while 10% GelMA retained a faint signal even at 4 weeks.^29^ Moreover, in terms of cell viability and proliferation, 10% GelMA performs better. Therefore, 10% GelMA may be the optimal choice for the construction of tissue‐engineered bladder patches and in vivo experiments.

Decellularization of BAM removes cellular and antigenic components that could cause immune reactions while maintaining the essential elements and three‐dimensional architecture of the native extracellular matrix.^40^ Studies suggest that BAM possesses bioinductive capabilities, promoting the development of functional tissues in vivo.^41^ Histological assessments revealed that BAM derived from bladder mucosa is inherently suited for bladder regeneration. The basal layer of BAM preserves the intact bladder mucosa, making it more compatible with the complex intravesical environment rich in urine. Its uneven and rough posterior surface enhances adhesion with hydrogels. Additionally, BAM exhibits exceptional mechanical properties, enduring deformations of up to 250% without substantial loss, thereby fulfilling the biomechanical demands of the bladder.^12^ Crucially, our tissue‐engineered bladder patches demonstrate exceptional stitchability, a critical feature for bladder repair in large animals, which has often been overlooked in previous studies on tissue engineering.^42^ As illustrated in Figure 4a,b, the increased bladder volume necessitates a greater number of sutures, often requiring dozens of stitches to prevent leakage. This places higher demands on the repair material. Previous studies primarily using small animal models have relied on scaffolds that may lack the toughness and stitchability required for human and large animal bladder repairs.^43^, ^44^, ^45^ Lawkowska highlights that due to the size discrepancy, biomaterials tested on small animal models may face significant challenges or even impracticality when applied to large animal or human trials.^46^ Current biomaterials, such as hydrogels, fail to meet this criterion and are often used as adhesives or carriers for cells or factors, emphasizing the need for basal repair material.^47^, ^48^ Thus, future research should prioritize the stitchability of patch materials, with BAM emerging as an ideal candidate.

In vivo implantation experiments demonstrated the extensive distribution of CM‐DiL‐labeled ADSCs across the hydrogel and BAM regions of the BGA patches, indicating successful survival and migration of ADSCs within the hydrogel and into the BAM post‐transplantation. A considerable number of unlabeled cells were observed in both patch types. The loose ECM structure of the hydrogel likely attracted a large number of cells to aggregate. Although both patch types exhibited cellular presence, the BGA patches showed superior vascularization, likely due to the induced differentiation of implanted ADSCs in vivo. This finding aligns with Gozde's study, which reported a threefold increase in vascularization in stem cell‐loaded hydrogels compared to non‐stem cell hydrogels.^49^ Furthermore, studies have indicated that hydrogels encapsulating growth factors can induce endogenous stem cells to migrate to damaged tissues and stimulate specific differentiation of the recruited cells. This can facilitate the regeneration of damaged tissues without stem cell transplantation, representing a potential direction for future research.^50^

The objective of tissue‐engineered patches is to preserve bladder morphology, regenerate bladder wall structures, and restore physiological functions. Postoperatively, the BGA group had a more abundant blood supply in the repair area and achieved better morphological remodeling, while the BG group experienced notable contracture due to the lack of vascularization. The BAM and natural bladder tissue in the BGA group were almost integrated, and more regenerative muscle bundles, blood vessels, and nerves were observed in the repair area, and the proliferation of collagen fibers was less than that in the BG group. These regenerated tissues with good vitality were beneficial for preserving better bladder capacity and compliance. These suggested ADSCs likely promote the regeneration of smooth muscle, blood vessels, and nerves, thereby improving bladder physiological functions.^13^, ^51^, ^52^ However, the PC group presented with severe scarring in the bladder healing area, where regenerated granulation tissue lacked normal elasticity, potentially affecting bladder capacity and compliance. By comparison with the experimental group, we found that this likely results from the effective reduction of collagen fiber deposition and prevention of pathological scar tissue formation, mediated by ADSCs and hydrogels through mechanisms such as inflammation modulation, fibroblast proliferation and activation regulation, and vascular reconstruction.^53^, ^54^ We also found that simple suture repair negatively impacted bladder capacity and compliance by over 40%, compared to the removal of 40% of tissue, indicating that the commonly used PC technique does not benefit bladder function preservation. While the BG group did not perform as well as the BGA group, it retained a degree of capacity and compliance, demonstrating the promising potential of tissue engineering techniques in revolutionizing bladder repair and reconstruction.

Driven by clinical needs, this study comprehensively evaluated the BGA bilayer patch, emphasizing its materials, cells, and in vivo performance. The study confirmed its remarkable reparative potential, focusing on functional and reparative properties rather than novel material development or material‐cell interaction mechanisms. The findings suggest that current advancements in this patch may already meet clinical requirements, positioning bladder tissue engineering on the cusp of clinical translation. Because the most recent clinical studies in 2018 and 2015 still relied on traditional PGA/PLGA scaffolds and amniotic membranes.^55^ While BAM, GelMA, and ADSCs may not be the most innovative materials and cells, their widespread availability and straightforward preparation make them ideal for large‐scale applications. Additionally, previous bladder tissue engineering studies often employed cell‐seeded scaffolds without hydrogel encapsulation. The approach of utilizing hydrogels for cell encapsulation may enhance clinical outcomes. By introducing a feasible and clinically promising bladder repair patch, this study aims to significantly benefit patients.

On the molecular level, some issues remain unresolved. Specifically, the influence and underlying mechanism of GelMA in inducing ADSCs differentiation into vascular or muscular lineages are unclear, as prior studies have shown that specific GelMA concentrations enhance the smooth muscle differentiation of mesenchymal stem cells^56^ Additionally, neuronal regeneration and the formation of neural innervation networks pose significant challenges, given the complexity of nerve regeneration during repair. A promising research direction involves achieving targeted stem cell differentiation within hydrogels to construct neuro‐vascular networks, ultimately modulating the contraction and relaxation of the regenerated bladder. The integration of novel elements and components into BAM and GelMA has the potential to yield more favorable outcomes.

## 4 MATERIALS AND METHODS

### 4.1 Synthesis and characterization of GelMA

#### 4.1.1 Synthesis of GelMA

To prepare a 10% (w/v) solution, 10 g of Type A gelatin derived from porcine (Sigma‐Aldrich, V900863, USA) was added to 100 mL of deionized water. The solution was heated and stirred at 50°C, then 1 mL of methacrylic anhydride (MA) (Sigma‐Aldrich, 276,685, USA) was slowly added dropwise over 30 min. The reaction continued for 3 h in the dark at 50°C. The reacted mixture was poured into a dialysis bag (MD44, 8000‐14000D) and dialyzed with deionized water at 50°C for approximately 7 days, with the water changed three times daily. After dialysis, the solution was frozen overnight at −80°C in an ultra‐low temperature freezer, followed by freeze‐drying for 2 days using a freeze dryer (Scientz‐10 N, China). The resulting dry, white, foamy GelMA was stored at −20°C for future use.

#### 4.1.2 Fourier Transform Infrared (FT‐IR) spectroscopy

Fourier transform infrared spectroscopy was performed to confirm the successful modification of GelMA. The prepared GelMA and gelatin samples were analyzed using a Fourier Transform Infrared Spectrometer (Bruker, INVENIO S, Germany). Data were collected with a resolution of 4 cm^−1^ and a wavenumber range of 500–4000 cm^−1^.

#### 4.1.3 Proton nuclear magnetic resonance ( ^1^H NMR) spectroscopy

The prepared samples of gelatin and GelMA were placed into separate EP tubes, with approximately 1 mL of deuterium oxide added to each. Ultrasonication was used to accelerate dissolution, and the samples were then transferred to nuclear magnetic resonance (NMR) tubes. A ^1^H NMR test was conducted using a 400/500 MHz Nuclear Magnetic Resonance spectrometer (Bruker, Avance NEO, Germany).

#### 4.1.4 Scanning Electron Microscope (SEM)

GelMA was dissolved in PBS and lithium phenyl‐2,4,6‐trimethylbenzoylphosphinate (LAP) (Sunp Biotech, SP‐BI‐C02‐2, China) to prepare 7.5%, 10%, and 12.5% (w/v) GelMA hydrogels, with LAP at a final concentration of 0.25% (v/v). For each group, 400 μL of GelMA hydrogel was dispensed into a 2 mL EP tube and exposed to 405 nm UV light for 1 minute. Following crosslinking, the hydrogels formed stable cylinders, which were subsequently frozen for 24 h each at −20°C and −80°C. After freeze‐drying the samples for 24 h, they were placed in liquid nitrogen to induce brittle fracture, obtaining cross‐sections. The samples were then metal‐coated and observed under a SEM at 200× magnification to examine their morphology and porosity.

### 4.2 Characterization of GelMA

#### 4.2.1 Swelling rate

For each GelMA group, 200 μL was crosslinked in EP tubes to form cylindrical samples. These samples were pre‐frozen for 24 h at −20°C and then at −80°C. After 24 h of freeze‐drying, the dry weights (Wd) were measured. The samples were then immersed in PBS buffer and removed at intervals of 3, 6, 12, 24, and 48 h. Surface water was gently wiped off with absorbent paper before weighing (Wt). Each group included 4 samples. The swelling ratio was calculated using the following formula:

$$\text{Swelling Ratio}\left(\%\right)=\frac{\mathrm{Wt}-\mathrm{Wd}}{\mathrm{Wd}}\times 100\%$$

#### 4.2.2 Degradation ratio

For each GelMA group, 400 μL was crosslinked in EP tubes to form cylindrical samples. After freezing and freeze‐drying, the dry weight (W0) was measured. The samples were then soaked in PBS buffer at 37°C for 2 days before being transferred to EP tubes. To each tube, 1 mL of PBS containing 2 U/mL of Collagenase II (LABLEAD, V0892, China) was added. At various time points (2, 4, 6, 8 h), the liquid was aspirated, and the remaining hydrogel samples were freeze‐dried. The dry weight after freeze‐drying (W1) was then measured. Each time point included three samples. The degradation ratio was calculated using the following formula:

$$\text{Degradation Ratio}\;\left(\%\right)=\frac{W0-W1}{W0}\times 100\%$$

#### 4.2.3 Mechanical properties

For each GelMA group, 400 μL was crosslinked in EP tubes to form cylindrical samples (5 mm height, 10 mm diameter). These samples underwent compression testing using a universal mechanical testing machine (Instron, 68SC‐1, USA), with a compression strain rate of 1 mm/min and a maximum strain of 70%. Young's modulus was determined from the stress–strain data. Four samples were tested per group. Additionally, a compression‐cycle test, consisting of 4 cycles at a maximum strain of 70%, was performed to observe any significant changes in the curve shape. Energy loss was calculated from the area under the loading‐unloading curves across the 4 cycles. The energy loss ratio was computed using the following formula:

$$\text{Energy loss ratio}\left(\%\right)=\begin{array}{l} \frac{\text{Area below loading curve}-\text{area below unloading curve}}{\text{Area below loading curve}}\times 100\% \end{array}$$

### 4.3 Preparation and characterization of BAM

#### 4.3.1 Preparation of BAM

A fresh porcine bladder, purchased from the market, was cleaned, and its mucosal layer was carefully separated using scissors. The intact mucosal tissue was placed in a plastic container, and 500 mL of 1% TritonX‐100 solution (Sigma‐Aldrich, T9284, USA) was added. The container was then placed on a shaking bed at 80 r/min for 8 h. The tissue was subsequently rinsed with deionized water for 20 min to remove residual TritonX‐100 solution. Next, 500 mL of 1% SDS solution (Sigma‐Aldrich, L4509, USA) was added, and the container was placed back on the shaking bed at 80 r/min for another 8 h. The tissue was rinsed again with deionized water for 20 min to eliminate any remaining decellularization solution, resulting in the BAM. The processed BAM was cut into 3 cm diameter circular pieces, frozen at −80°C overnight, freeze‐dried for 24 h, and stored at −20°C.

#### 4.3.2 Histological staining of BAM

Both natural bladder mucosa and BAM were fixed by soaking in a 4% paraformaldehyde solution, dehydrated with an ethanol gradient, and embedded in paraffin. The 4 μm sections were stained with H&E and DAPI to evaluate nuclear residues. Masson's trichrome staining was performed to observe collagen distribution.

#### 4.3.3 Tensile testing of BAM

Bladder mucosa and BAM were cut into strips measuring 3 cm in length, 1.5 cm in width, and 1 mm in thickness. These strips were placed on a universal mechanical testing machine for tensile testing, with the gap between fixtures set to 20 mm and a testing speed of 50 mm/min, stretching until rupture. Tensile stress, fracture strain, and Young's modulus were calculated from the stress–strain data. Each group included four samples.

#### 4.3.4 Suture cutting resistance experiment

Samples of the same dimensions were prepared, with a 5–0 absorbable suture passed through the center of each. One end of the patch was fixed to the upper part of the universal mechanical testing machine, while the suture was secured to the lower part. Tensile testing was conducted with a fixture gap of 30 mm and a constant testing speed of 50 mm/min. The test continued until the suture cut through the sample, allowing for the calculation of the maximum tensile force. Each group included four samples.

#### 4.3.5 DNA quantitative detection of BAM

Equal weights (50 mg) of normal bladder tissue (*n* = 3) and BAM (*n* = 3) were cut into tissue suspensions. DNA was then extracted and purified by centrifugation following the instructions in the Genomic DNA Kit (Tiangen Biotech, DP304‐02, China). Finally, the extracted DNA was quantitatively analyzed using a Nanodrop spectrophotometer (Thermo Fisher Scientific, ND3300, USA).

### 4.4 Biocompatibility of GelMA and BAM

#### 4.4.1 Extraction and culture of ADSCs

Inguinal adipose tissue (approximately 2 × 2 cm) was collected from a beagle dog under anesthesia and thoroughly cleaned with PBS containing 1% penicillin–streptomycin (Gibco, 15,140,122, USA). The adipose tissue was then minced, and 15 mL of DMEM, 12 mL of 0.25% trypsin, and 3 mL of type I collagenase (Invitrogen, 17,100,017, USA) was added. The mixture was digested on a magnetic stirrer at 300 r/min and 37°C for 30 min. Digestion was terminated by adding an equal amount of DMEM medium containing 10% FBS, and the solution was filtered through a 200‐mesh screen. The filtrate was centrifuged at 1200 rpm for 10 min. After discarding the supernatant, the cells were resuspended in a canine adipose‐derived mesenchymal stem cell complete medium (OriCell, CAXMD‐90011, USA) and cultured in a 37°C incubator with 5% CO_2_. The medium was replaced every 2 days. Flow cytometry with antibodies CD29, CD34, and CD45 was used for the identification of the P3 generation (Figure S2).

#### 4.4.2 Cell viability in GelMA

Cell viability was evaluated using the LIVE/DEAD™ Cell Viability Assay Kit (Invitrogen, L3224, USA). The hydrogel was sterilized by filtration through a 0.22 μm filter. ADSCs from the P3‐P5 generations were collected and mixed with three groups of GelMA at a density of 1 × 10^7^ cells/mL. For each group, 100 μL of the hydrogel mixture was evenly dispensed onto a glass slide and exposed to 405 nm UV light for 40 s at an intensity of 80 mW/cm^2^. The slides were transferred to a 24‐well plate and incubated with 500 μL of culture medium. After 1, 4, and 7 days of incubation, the medium was removed, and the slides were washed twice with PBS. Subsequently, 500 μL of PBS, 1 μL of Component B (dead cell stain, red), and 0.25 μL of Component A (live cell stain, green) were added, followed by 20 min of incubation at 37°C in the dark. The slides were then transferred to a small dish and observed under a laser‐scanning confocal microscope (Zeiss, LSM900, Germany). Cell viability was calculated by dividing the number of live cells by the total number of cells.

#### 4.4.3 Cell proliferation in GelMA

The Cell Counting Kit‐8 (LABLEAD, CK001, China) assessed cell proliferation within the hydrogel. ADSCs from the P3‐P5 generations were mixed with three groups of GelMA at a density of 1 × 10^7^ cells/mL. The hydrogel mixtures were seeded into a 96‐well plate at 10 μL per well. After photocrosslinking, a complete medium was added. Following 1, 4, and 7 days of incubation, the medium was removed, and the wells were washed twice with PBS. Subsequently, 100 μL of DMEM medium and 10 μL of CCK‐8 staining solution were added, and the plate was incubated at 37°C for 2 h. Absorbance was measured at 450 nm using an MD microplate reader (Molecular Devices, VersaMax, USA). The optical density (OD) value, directly proportional to the number of live cells, was recorded. Each group was tested in triplicate.

#### 4.4.4 Cytoskeleton staining

After a 7‐day culture of GelMA‐ADSCs bio‐ink on glass slides, the samples underwent two PBS washes and fixation with 4% paraformaldehyde at room temperature for 40 min, followed by two additional PBS washes. The cells were then permeabilized with 0.1% Triton X‐100 (Beyotime, P0096, China) for 40 min. Post‐permeabilization, the samples were washed twice with PBS and blocked using a protein‐blocking buffer (Abcam, ab64226, USA) for 30 min. Actin staining was carried out with SF488 Phalloidin (G‐Clone, CS0143‐300 T, China) for 60 min, followed by DAPI staining (Invitrogen) for the nuclei. Observation of the samples was conducted using a laser‐scanning confocal microscope.

#### 4.4.5 Biocompatibility of BAM

Following irradiation sterilization, BAM was soaked in canine ADSCs complete medium for 48 h, after which the extract was collected. ADSCs at passages 3–5 were harvested and mixed with 10% GelMA hydrogel at a density of 1 × 10^7^ cells/mL. BAM extract served as the experimental group, while the complete medium acted as the control group. Live/dead staining, CCK‐8 assay, and cytoskeleton staining were conducted as described in Sections 4.4.2, 4.4.3, and 4.4.4.

#### 4.4.6 RT‐PCR analysis

P5 ADSCs were resuspended in GelMA of different concentrations at a density of 1 × 10^7^ cells/mL after digestion and centrifugation. These suspensions were then added to a 12‐well plate at 300 μL per well, with three wells per group, followed by 40 s of 405 nm UV exposure. The medium changes every 2 days and the hydrogels were lysed by GelMA lysate (SunP Biotech, SP‐BI‐G04‐1, China) on Day 7. The ADSCs were collected by centrifugation, and RNA was extracted using Trizol reagent (Invitrogen, USA). The RNA was reverse‐transcribed into cDNA by the PrimeScript RT Kit (Takara, RR036A, Japan). Gene expression levels of Oct4 and Sox2 were analyzed by RT‐PCR using the LightCycler 96 system (Roche, Switzerland) with a TB Green Primix Kit (Takara, RR820A, Japan). The primer sequences are provided in Supplementary Data Table S2.

### 4.5 Construction of BAM‐GelMA (BG) bilayer patch

To prepare the BG patch, 1 mL of 10% GelMA was uniformly dropwise added to a 3 × 3 cm BAM patch and irradiated with 405 nm UV light (80 mW/cm^2^) for 40s to cross‐link GelMA and tightly fit with BAM.

#### 4.5.1 SEM of BG bilayer patch

A segment of the BG bilayer patch was then stored in −20°C and − 80°C freezers for 24 h each, followed by a 24‐h freeze‐drying process. The freeze‐dried sample was subsequently brittle and fractured in liquid nitrogen to obtain the cross‐section. After metal coating, the samples were examined under a SEM at 100× and 1000× magnifications to observe the morphology of the cross‐sections.

#### 4.5.2 In vivo culture of BAM‐GelMA‐ADSCs (BGA) bilayer patch

P3 ADSCs were resuspended in 1 mL of PBS, followed by the addition of 3 μL of CM‐DiL staining solution (Invitrogen, C7000, USA). The cells were incubated at 37°C in a 5% CO_2_ incubator for 5 min, then transferred to a 4°C environment for an additional 15 min. After centrifugation at 1000 rpm for 5 min and removal of the supernatant, 1 mL of GelMA was mixed with the ADSCs (cell density: 1 × 10^7^/mL). The hydrogel was then applied onto BAM to construct a BGA bilayer patch.

Both BGA and BG patches were cultured in a medium for 7 days before implantation into the abdominal cavity of beagles (3 patches in 2 beagles each). The patches were secured to the greater omentum with 6–0 absorbable sutures, ensuring hydrogel contact with the omentum. After 7 days, the patches were removed and washed with physiological saline to eliminate blood stains. Fixation of the patches in 4% paraformaldehyde for 48 h was followed by ethanol gradient dehydration, paraffin embedding, and sectioning. Sections (4 μm) were prepared for H&E staining and immunofluorescence staining. For immunofluorescence staining, incubation with the primary antibody PECAM1 (1:200, MAB11349, Abnova) at 4°C overnight was conducted. Subsequently, the sections were incubated with a species‐matched secondary antibody, and nuclei were counterstained with DAPI (antibody information: Table S3). Images were captured using a pathology slide scanner (3D HISTECH, Pannoramic MIDI, Hungary). Image analysis using Image J (Version 1.53) involved examining six fields of view from three randomly selected slides to quantify PECAM1‐positive blood vessels.

### 4.6 Animal grouping and surgery

#### 4.6.1 Beagles grouping

Eighteen female beagle dogs (aged 8–10 months, weighing 10–12 kg) were randomly divided into three groups: the BGA group, the BG group, and the PC group. Urodynamic testing was performed on all animals before surgery. At 4 and 12 weeks post‐surgery, three dogs from each group were randomly selected for bladder CT scans, cystoscopy, urodynamic testing, and histological examination. The female beagle dogs used in the experiment were sourced from the Animal Experiment Center of the PLA General Hospital. The Ethics Committee of the PLA General Hospital approved the experiment (No. 2023‐X19‐37).

#### 4.6.2 Bladder reconstruction

After anesthetizing the beagle, an 8F catheter was inserted to drain the urine and inject 50 mL of sterile saline. The lower abdomen was sterilized, and an incision was made to expose the distended bladder. Forty percent of the tissue at the apex was resected, taking care to avoid damage to the bladder trigone. The bladder defects were repaired using BGA patches and BG patches, respectively. The boundaries of the defect area were marked with four non‐absorbable 3–0 suture threads, and the patches and bladder were sutured intermittently with absorbable 5–0 sutures. In the PC group, the resected tissue ends were sutured together. Sterile saline was infused into the reconstructed bladder through the catheter to confirm its tightness, and the incision was sutured layer by layer. Postoperatively, ceftriaxone sodium was administered subcutaneously at a dose of 0.5 g/day for 7 days to prevent infection.

### 4.7 Postoperative imaging examination

#### 4.7.1 Bladder CT examination

Following the beagle's anesthesia, an 8F catheter was inserted to drain the urine. A mixture of 20 mg diatrizoate meglumine contrast agent and 0.9% normal saline at a 1:2 ratio was then prepared. This diluted contrast agent was injected into the bladder through the catheter until it drained from the urethra. The injection was stopped and the catheter tip was clamped. A spiral CT scan (United Imaging, uCT 760, China) provided three‐dimensional reconstructed images and plain radiographs of the bladder.

#### 4.7.2 Cystoscopy

Cystoscopy on the beagle dogs utilized a single‐use digital flexible ureteroscope (ZebraScope, HUS‐T101, China). A 10F endoscope was inserted into the bladder via the urethra, and the bladder was filled with 0.9% normal saline to enhance observation.

### 4.8 Urodynamic examination

Urodynamic testing was conducted using a urodynamic testing device (Laborie, Aquarius TT Triton, Canada). A 10F urodynamic anal manometry catheter was inserted into the rectum, and an 8F urodynamic double‐lumen manometry catheter was inserted into the bladder. The 8F catheter was connected to 0.9% normal saline, with the bladder continuously infused at a rate of 10 mL/min for filling cystometry. Infusion ceased when continuous urine outflow was observed at the urethral orifice, indicating the onset of urination. Bladder volume and bladder compliance were recorded during urination. Each beagle underwent two recorded urination cycles.

### 4.9 Histological examination

Tissue samples were collected at 4 and 12 weeks postoperatively. The bladder was cleaned with 0.9% normal saline, fixed in 4% paraformaldehyde, dehydrated in graded ethanol, and embedded in paraffin for sectioning. Sections (4 μm) were stained using H&E, Masson, and immunofluorescence techniques. For immunofluorescence staining, primary antibodies AE1/AE3, α‐SMA, β‐III Tubulin, and PECAM1 were used for incubating sections overnight at 4°C (antibody information: Table S1). Subsequently, sections were treated with species‐matched secondary antibodies and counterstained with DAPI to visualize nuclei. Images were captured with a pathology slide scanner (3D HISTECH, Pannoramic MIDI, Hungary). Fluorescence intensity analysis, including the quantification of α‐SMA, β‐III Tubulin, and PECAM1‐positive blood vessels, was performed on six fields of view randomly selected from three slides using ImageJ (Version 1.53), to evaluate bladder tissue regeneration in the repaired and replaced areas of each group.

### 4.10 Statistical analysis

Quantitative data are presented as mean ± standard deviation (SD). ANOVA was employed for comparisons among multiple groups, while the paired *t*‐test was used for comparisons between two groups. Statistical significance was set at *p* < 0.05. All statistical analyses were conducted using GraphPad Prism 9.0 software.

## 5 CONCLUSION

GelMA served as a cellular carrier for ADSCs in this study, with its optimal concentration identified. Using BAM as the basal material, the BAM‐GelMA‐ADSCs tissue‐engineered bladder bilayer patch was developed. This patch demonstrates excellent mechanical properties and biocompatibility, making it suitable for clinical manipulation and suturing. ADSCs within the patch facilitate rapid vascularization of the graft post‐implantation. Animal experiments show that this patch enhances muscle, vascular, and neural regeneration in the repair area, leading to superior morphological and functional restoration. In summary, these BAM‐GelMA bilayer patches loaded with ADSCs are suitable for repairing large‐scale bladder defects and hold significant clinical application potential.

## AUTHOR CONTRIBUTIONS

**Ziyan An:** Conceptualization, Methodology, Formal analysis, Data curation, Visualization, Writing – original draft. **Pengchao Wang:** Conceptualization, Methodology, Data curation, Writing – original draft. **Zhengyun Ling:** Conceptualization, Methodology, Formal analysis. **Kaipeng Bi:** Methodology, Investigation, Formal analysis. **Zheng Wang:** Methodology, Software, Visualization. **Jinpeng Shao:** Methodology, Investigation. **Jian Zhao:** Methodology, Investigation. **Zhouyang Fu:** Methodology, Investigation. **Meng Huang:** Methodology. **Wenjie Wei:** Methodology. **Shuwei Xiao:** Conceptualization, Supervision, Writing – review & editing. **Jin Zhou:** Conceptualization, Supervision, Writing – review & editing. **Weijun Fu:** Project administration, Supervision, Writing – review & editing, Funding acquisition.

## FUNDING INFORMATION

This research was supported by grants from the National Natural Science Foundation of China (Grant number 82270721) and the Beijing Natural Science Foundation (Grant number 7252169).

## CONFLICT OF INTEREST STATEMENT

The authors have no relevant financial or non‐financial interests to disclose.

## Supporting information


**Data S1:** Supporting Information.

## Data Availability

The data sets used and analyzed during the current study are available from the corresponding author upon reasonable request.
