# Supplementary material for: The BAM‐GelMA‐ADSCs bilayer patch promotes tissue regeneration and functional recovery after large‐area bladder defects in beagles
Source: Bioeng Transl Med. 2025 Jan 15;10(3):e10745. doi: 10.1002/btm2.10745 (PMC12079509; doi:10.1002/btm2.10745)
Supplement: Supplementary file 1 — Data S1: Supporting Information. [file BTM2-10-e10745-s001.docx]

**The BAM-GelMA-ADSCs bilayer patch promotes tissue regeneration and functional recovery after large-area bladder defects in beagles**

**Authors:** Ziyan An ^1,2,3^, Pengchao Wang ^4^, Zhengyun Ling ^5^, Kaipeng Bi ^1,5^, Zheng Wang ^1,2^, Jinpeng Shao ^1,2^, Jian Zhao ^6^, Zhouyang Fu ^7^, Meng Huang ^2^, Wenjie Wei ^1,2^, Shuwei Xiao ^8^, Jin Zhou ^9^, Weijun Fu ^1,3^

Ziyan An, Pengchao Wang and Zhengyun Ling contributed equally to this study.

**Corresponding authors:**

Weijun Fu, Department of Urology, Third Medical Center, PLA General Hospital, Yongding Road No. 69, Beijing 100039, China.

E-mail address: fuweijun@hotmail.com.

Jin Zhou, Beijing Institute of Basic Medical Sciences, Taiping Road No.27, Beijing 100850, China.

E-mail address: sisun819@outlook.com.

Shuwei Xiao, Department of Urology, Air Force Medical Center, Fucheng Road No.30, Beijing, 100142, China.

E-mail address: xiaoshuwei00@163.com.


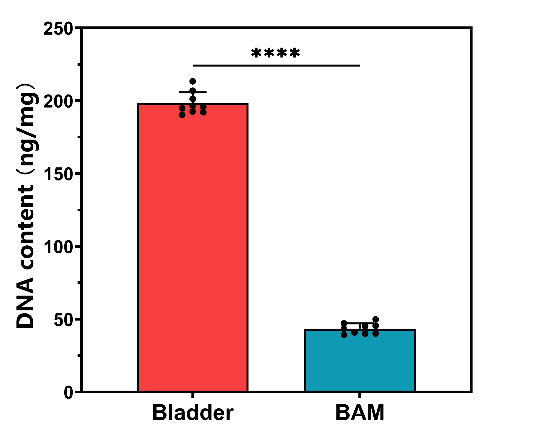


**FIGURE. S1. DNA quantitative detection of BAM (n=3) (*P* values are calculated using the paired *t* test, *****P* < 0.0001).**


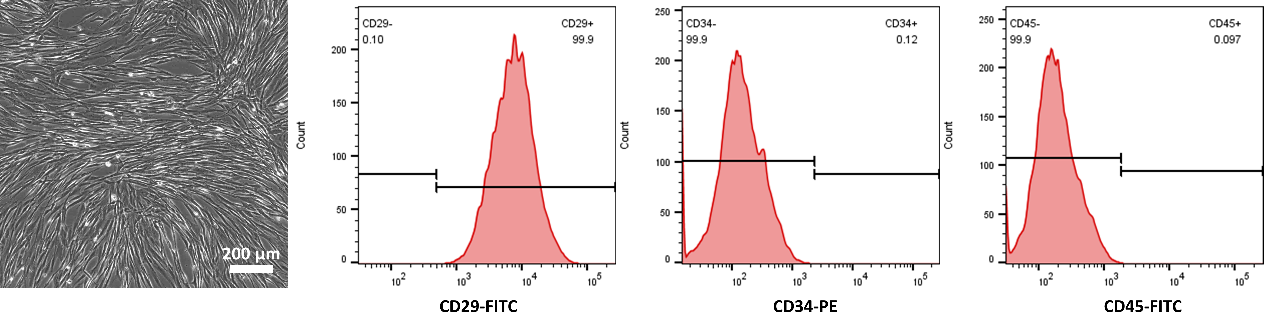


**FIGURE. S2. Flow cytometry identification of ADSCs.**


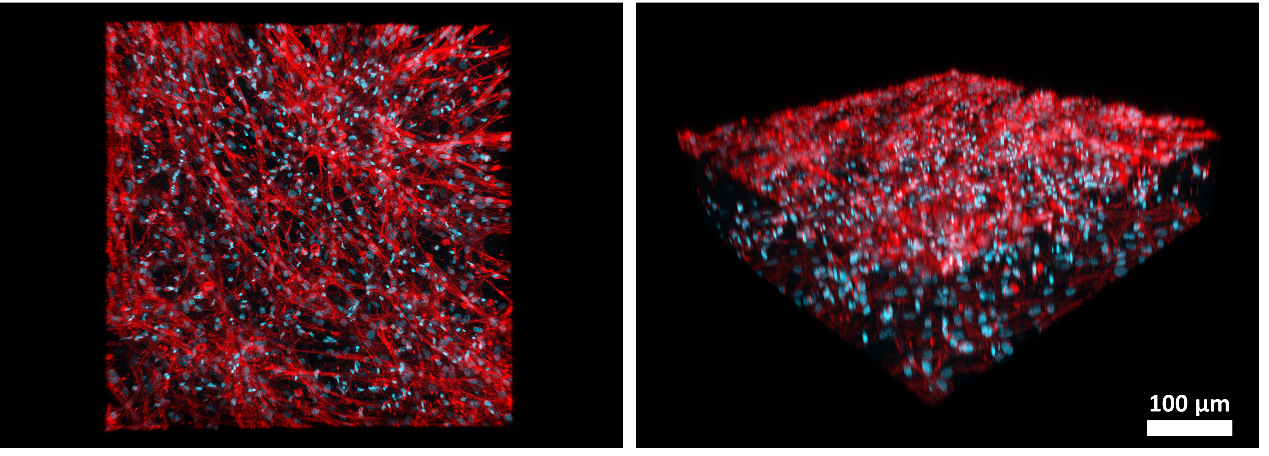


**FIGURE. S3. Cytoskeleton staining of ADSCs in 10% GelMA.**


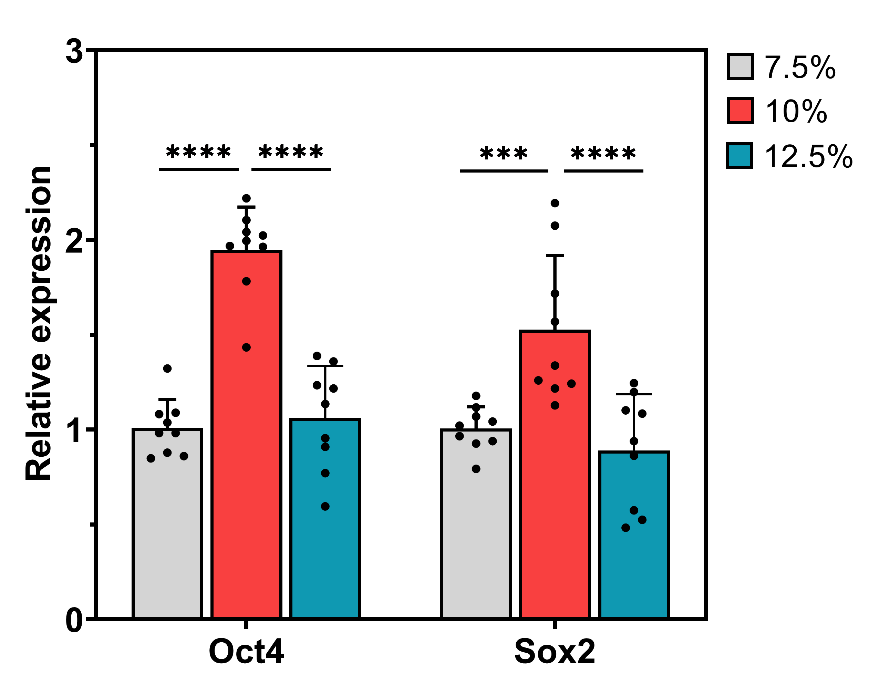


**FIGURE. S4. The mRNA expression levels of Oct4 and Sox2 of ADSCs cultured in different concentrations of GelMA for 7 days *in vitro* (n=3)** **(*P* values are calculated using the two-way ANOVA, ****P* < 0.001, *****P* < 0.0001).**

**TABLE S1. Detailed antibodies information for flow cytometry**

| **Against** | **Brand** | **Article number** | **Dilution** |
| --- | --- | --- | --- |
| CD29-FITC | BD Biosciences | BD561796 | 1:100 |
| CD34-PE | Genetex | GTX75414 | 1:100 |
| CD45-FITC | BD Biosciences | BD561867 | 1:100 |

**TABLE S2. Primer sequences for RT-PCR amplification of target genes**

| **Gene** | **Primer** |
| --- | --- |
| GAPDH-forward | 5’-TCCACGGCACAGTCAAGGC-3’ |
| GAPDH-reverse | 5’-AACATACTCAGCACCAGCATCAC-3’ |
| Oct4-forward | 5’-AGAACCGAGTGAGAGGCAACC-3’ |
| Oct4-reverse | 5’-GCTGGGCAATGTGGCTGATC-3’ |
| Sox2-forward | 5’-GAACGCCTTCATGGTGTGGTC-3’ |
| Sox2-reverse | 5’-GCTTGCTGATCTCCGAGTTGTG-3’ |

**TABLE S3. Detailed antibodies information of primary and corresponding secondary antibodies**

| **Primary**  **antibodies** | **Brand** | **Article**  **number** | **Dilution** | **Secondary**  **antibodies** | **Brand** | **Article**  **umber** | **Dilution** |
| --- | --- | --- | --- | --- | --- | --- | --- |
| AE1+AE3 | NOVUS | NBP2-29429 | 1:200 | Goat Anti-Mouse  (HRP) | Servicebio | GB23301 | 1:500 |
| α-SMA | Abcam | ab184675 | 1:100 | - | - | - | - |
| PECAM1 | Abnova | MAB11349 | 1:200 | Goat Anti-Mouse  (Alexa 488) | Servicebio | GB25301 | 1:300 |
| β-III  Tubulin | Abcam | ab215037 | 1:500 | Goat Anti-Rabbit  (Cy3) | Servicebio | GB21303 | 1:300 |
